# What factors affect team members’ evaluation of collaboration in medical teams?

**DOI:** 10.3389/fpsyg.2022.1031902

**Published:** 2023-01-12

**Authors:** Juliane E. Kämmer, Simone Ehrhard, Olga Kunina-Habenicht, Sabine Weber-Schuh, Stefanie C. Hautz, Tanja Birrenbach, Thomas C. Sauter, Wolf E. Hautz

**Affiliations:** ^1^Department of Emergency Medicine, Inselspital, University Hospital Bern, University of Bern, Bern, Switzerland; ^2^Psychological Assessment, TU Dortmund University, Dortmund, Germany

**Keywords:** collaborative decision making, perceived teamwork quality, healthcare teams, structural equation modeling, supervisor–trainee relationships

## Abstract

**Introduction:**

Perceived teamwork quality is associated with numerous work-related outcomes, ranging from team effectiveness to job satisfaction. This study explored what situational and stable factors affect the perceived quality of teamwork during a specific team task: when a medical team comprising a senior (supervisor) and a junior (trainee) physician diagnoses a patient.

**Methods:**

During a field study in an emergency department, multisource data describing the patients, the diagnosing physicians, and the context were collected, including physicians’ ratings of their teamwork. The relationships between perceived teamwork quality and situational (e.g., workload) and stable (e.g., seniority) factors were estimated in a latent regression model using the structural equation modeling (SEM) approach.

**Results:**

Across the *N* = 495 patients included, SEM analyses revealed that the patient-specific case clarity and urgency influenced the perceived teamwork quality positively, whereas the work experience of the supervisor influenced the perceived teamwork quality of both supervisor and trainee negatively, albeit to different degrees.

**Discussion:**

Our findings shed light on the complex underpinnings of perceived teamwork quality, a performance-relevant factor that may influence work and organizational effectiveness in healthcare settings.

## 1. Introduction

Teamwork has been repeatedly identified as the number one global workforce trend, spanning domains such as law, health care, engineering, and science (Edmondson, 2012; Deloitte Insights, 2019). In companies, for example, multidisciplinary or cross-functional teams often collaborate during product development and innovation projects (Hoegl and Praven Parboteeah, 2003; Dayan and Di Benedetto, 2009). In health care, teamwork occurs across the continuum of medical care, such as when a team of health-care professionals together with the patient and their family engage in finding the correct diagnosis or deciding on a treatment (Committee on Diagnostic Error in Health Care et al., 2015). Reasons for this trend include increased specialization, constant changes in work environments, and increasingly complex problems that cannot be solved by single experts alone but instead demand cross-disciplinary and interprofessional collaboration. It is therefore not surprising that team performance is linked to organizational success (Hoegl and Gemuenden, 2001; Dayan and Di Benedetto, 2009; Manser, 2009; Kozlowski and Bell, 2013; Schmutz and Manser, 2013). Yet, where collaboration and coordination are crucial, failures in teamwork can have detrimental consequences. For example, communication breakdown is one of the most common causes of adverse events in medicine, including diagnostic errors (Risser et al., 1999; Manser, 2009). Diagnostic error is a particularly common, enormously harmful, and extremely costly type of medical error, constituting not only an individual but also a societal burden (Hautz et al., 2019; Hautz, W. E. et al., 2020).

Research on the determinants of successful teamwork has flourished over the last decades (Mathieu et al., 2017) and has proven its relevance to organizational performance by, for example, informing the development of theory-based team trainings (Salas et al., 2008) or identifying successful coordination behaviors (Schmutz et al., 2015). Following established taxonomies (e.g., Hoegl and Gemuenden, 2001; Salas et al., 2005), we define teamwork as a higher order construct encompassing a number of different facets such as coordination, communication, and leadership. *Objective* team performance can be distinguished from *subjective* teamwork quality, but these aspects are correlated such as when better perceived teamwork quality is related to better quality of care (Manser, 2009; Berry et al., 2020). Whereas objective team performance can be assessed by trained raters who observe team behaviors (e.g., Kolbe and Boos, 2019); subjective/perceived teamwork quality can be assessed by asking team members about their perceptions or evaluations (Hoegl and Gemuenden, 2001). In this study, we focused on *perceived* teamwork quality for two reasons. First, eliciting team perceptions is an efficient means of gaining insight into teamwork when the assessment of teamwork through observations is impractical or impossible; second, team perceptions are also a team outcome in their own right, which may in turn influence more distal variables (Mathieu et al., 2008). For example, favorable perceptions of teamwork have been shown to be positively related to job satisfaction and well-being, as well as reduced staff turnover (Dechairo-Marino et al., 2001).

An important finding of research into perceived teamwork quality is that perceptions of teamwork vary with position in the organizational hierarchy (Hautz, S. C. et al., 2020), seniority (Fleming et al., 2006), discipline (Ummenhofer et al., 2001), and professional group (Temkin-Greener et al., 2004). For example, studies in health care have found that physicians consistently rate the quality of teamwork higher than nurses do (Flin et al., 2006; Makary et al., 2006; Wauben et al., 2011; Tang et al., 2013; Müller et al., 2018). Yet, despite the empirical evidence of divergent teamwork perceptions, only a few studies have examined the underlying reasons for the observed differences. Possible reasons that have been discussed include that expectations (Frasier et al., 2017), communication styles (Jones and Durbridge, 2016), and stereotypes (Lingard et al., 2005; Kämmer and Ewers, 2021) vary with roles and profession—and hence shape perceptions. In addition, even though teams have a shared team goal, subtasks likely vary by role and profession and may thus influence the perceived strain (Keller et al., 2021) and perspective on the overall teamwork quality. For example, in a study of team performance in the emergency room, residents (i.e., junior doctors) were found to feel particularly stressed (Ummenhofer et al., 2001); similarly, trainee surgeons were particularly affected by tension in their team’s communication (Lingard et al., 2002).

Most of these reasons pertain to rather stable person-specific factors (e.g., position in the organizational hierarchy, seniority; Müller et al., 2018); less is known about situational factors that affect the perception of teamwork during a specific team event. Previous research suggests that perceptions of teamwork quality in general may be influenced by factors other than perceptions of teamwork quality in a specific team event, resulting in differences between general survey studies and studies of single team events (Müller et al., 2018). Also, from an organizational perspective, situational factors are particularly interesting because they are partially modifiable and can be changed if necessary.

The purpose of this study was thus to explore what situational and stable factors affect the perceived quality of teamwork during a specific team task, that is, when diagnosing and treating a single patient. One reason for investigating situational factors is the practical implication: Whereas stable factors such as hierarchy and seniority cannot be influenced, situational factors such as noise are (partially) modifiable and can be changed if they turned out to have a detrimental effect on teamwork or performance. Another reason is that there is an imbalance in the evidence base concerning which factors impact teamwork perceptions, lacking the variety of possible influencing factors such as situation-specific ones. Therefore, we conducted an exploratory analysis of data collected during a field study in an emergency department (ED), where we obtained ratings of teamwork quality from the medical team members for each patient they diagnosed and treated (Hoegl and Gemuenden, 2001). The ED is a task-oriented environment, in which team members share a common goal (i.e., treating a patient) and work highly interdependently in *ad-hoc* teams.

We focus on the core-team of senior/attending physician and junior/resident physician who work together in an apprenticeship model as supervisor (i.e., senior physician) and trainee (i.e., junior physician), a constellation that is common in many educational settings in the workplace. The trainee usually attends to a patient first, takes the patient’s history, conducts a physical exam and orders initial diagnostic tests before reporting to the supervising fully licensed physician. Together, they then analyze available diagnostic test results and decide on additional tests before ultimately settling on a diagnosis and initiating treatment. Of course, this general procedure varies in accordance with numerous factors including the urgency of the patient’s condition and the trainee’s skills.

In sum, we had two research questions: (1) Do supervisors and trainees differ in their perceptions of teamwork quality? (2) What factors influence team members’ perceptions of teamwork quality? To obtain a comprehensive picture of factors that might impact the individual team member’s perceptions, we collected data on various variables that count as “input” factors in classic input–process–output models of team effectiveness (McGrath, 1964; Ilgen et al., 2005; Mathieu et al., 2008), including patient, physician, and context factors (Durning et al., 2012).

## 2. Materials and methods

### 2.1. Study design

This study is an exploratory secondary analysis of a data set obtained in the cDx (change in diagnosis) study, a prospective, observational cohort study of diagnostic decision making in the ED of a university-affiliated tertiary care hospital in Switzerland (Hautz et al., 2016a, 2019). In this study, data on patients, physicians, and context factors were prospectively collected for all non-vitally threatened ED patients aged 18 or older who were hospitalized from the ED to any internal medicine ward in a 4-month period.

The ED where the study took place is a self-contained interdisciplinary unit and sees more than 45,000 patients each year (Exadaktylos and Hautz, 2015). This study setting was chosen for three reasons: (1) We aimed at including healthcare teams with a diverse level of acquaintance (from well-known to completely new), which is more likely to occur in larger hospitals; (2) we wanted to make sure that collaboration happened face-to-face, which effectively requires larger settings where senior physicians are physically present around the clock; and (3) we expected to achieve a larger participation rate because both physicians and patients in a university-affiliated hospital are more used to taking part in research projects and would thus be more willing to participate in the study.

### 2.2. Data collection

Prior to patient recruitment, all ED physicians were invited to participate in the study and asked to provide demographic and professional data including on age, gender, extent of work experience (i.e., years of work experience since graduation and in emergency medicine in particular), professional background (specialization, e.g., internal medicine), and current position (i.e., junior or senior physician). After admission of each patient to a medical ward, the treating ED junior and senior physician were asked to individually fill in a questionnaire that enquired about physician factors (i.e., confidence in diagnosis, familiarity with similar cases, ease of the diagnostic process), patient factors (atypical/typical presentation), and our dependent variable, teamwork quality (frequency of collaboration in the past and quality of collaboration with the other physician during this case). All items fit on a single page to help in obtaining a high response rate (see Table 1; Hautz et al., 2016b for original questionnaires). We employed a code-generation instruction on every questionnaire that ensured that all questionnaires could be associated with the person who filled it in while at the same time protecting the respondents’ anonymity. Participating physicians received compensation of 10 Swiss francs (approximately $10.05 at the time of data collection) for each completed questionnaire.

**Table 1:** Overview of collected data and how they were summarized into latent factors.

Factor	Measure	Values	Latent factor in the SEM
Physician factor	Age	Number of years	Physician professional experience
	Work experience (total)	Number of years	
	Work experience in ED	Number of years	
	Confidence in diagnosis	1 *unconfident* – 5 *confident*	Case clarity
	Familiarity with similar cases	1 *never encountered* – 5 *familiar*	
	Ease of the diagnostic process	1 *difficult* – 5 *easy*	
	Perceived presentation of patient	0 *atypical*, 1 *typical*	
Patient factor	Triage category	1 *treated immediately by a physician*	Case urgency
		2 *treated within 20 min by a physician*	
		3 *treated within 120 min by a physician*	
		4 *not an urgent treatment situation*	
		5 *follow-up check*	
	Treated in resuscitation bay	0 *not in resuscitation bay*, 1 *in resuscitation bay*	
	Mortality	0 *alive*, 1 *dead*	
Context factor	Noise (SD)	Standard deviation of noise in intervals of 15 min, averaged across the time the patient spent in the ED	Objective workload
	Noise (max)	Peak noise in intervals of 15 min, averaged across the time the patient spent in the ED	
	Objective workload during diagnostic process (SD)	Standard deviation of NEDOCS in intervals of 15 min, averaged across the time the patient spent in the ED	
	Objective workload during diagnostic process (max)	Maximum value of NEDOCS in intervals of 15 min, averaged across the time the patient spent in the ED	
Team factor	Perceived quality of collaboration with the other physician	1 *was alone* – 5 *very good*	Quality of teamwork (dependent variable)
	Frequency of collaboration with the other physician in the past	1 *rarely* – 5 *very often*	

ED, emergency department; NEDOCS, National Emergency Department Overcrowding Scale (Weiss et al., 2004); SEM, structural equation modeling.

Patients’ medical data (e.g., triage, treatment in resuscitation bay) were extracted from the ED’s electronic health record. The latent factor for the objective workload was measured by indicators for noise and the National Emergency Department Overcrowding Scale (NEDOCS; Weiss et al., 2004). The level of noise was measured by continuously logging noise levels in decibels at the physicians’ workplace in the ED with a sound meter (HD600, Extech Instruments, Nashua, New Hampshire). The objective workload was measured with the NEDOCS in intervals of 15 min (for details see Weiss et al., 2004, 2006). Across each patient’s length of stay in the ED, the respective average and peak noise and NEDOCS levels were calculated.

### 2.3. Statistical analyzes

Descriptive analyzes were conducted with R software for statistical computing (Version 4.1.1) and IBM SPSS (Version 21). Structural equation modeling (SEM) analyzes were conducted with the statistical software Mplus 8.0 (Muthén and Muthén, 1998, 2010) using the weighted least square mean and variance adjusted (WLSMV) estimator, which was developed for categorical and ordinal indicator variables. As model fit indices, we report the comparative fit index (CFI) and root-mean-square error of approximation (RMSEA) in addition to the χ^2^ value. These measures of fit were included because the χ^2^ value depends on sample size, where even small amounts of misfit can lead to significant χ^2^ values when sample sizes are moderate to large (Chen, 2007). As a rule of thumb, a ratio of the χ^2^ value to the number of degrees of freedom smaller than 2 indicates a good model fit. For RMSEA, values smaller than 0.05 reflect a good fit and values between 0.05 and 0.08 an adequate fit. For CFI, values of 0.90 or higher are considered a satisfactory fit, and values above 0.95 are considered an excellent fit (Hu and Bentler, 1999). Missing data were considered during the model estimation as a default option in Mplus by using the WLSMV estimator that uses pairwise present data (Asparouhov and Muthen, 2010).

In SEM, we postulated a latent regression model with several latent factors (Table 1): *physician professional experience*, *case clarity*, *case urgency*, *workload*, and *perceived teamwork quality*. Age and postgraduate experience overall and in emergency medicine specifically were used to generate the latent factor *professional experience*. The latent factor *case clarity* was modeled using the indicators diagnostic confidence, familiarity with symptoms, perceived ease of the diagnostic process, and whether the patient presentation was perceived as typical or atypical. The latent factor *case urgency* was modeled using the indicators triage category, treatment in a resuscitation bay, and mortality of the patient. *Objective workload* was captured in a separate latent factor by considering the standard deviation and maximum values of the NEDOCS and noise level at the physician’s workstation. We included both measures because it is unclear whether it is the peak load that most impairs performance or whether it is the load variation. To account for both options, both variables were included as indicators into the model. The latent factor *perceived teamwork quality* was modeled by the indicators quality of interaction and quantity of collaboration (Table 1). Because familiarity fosters teamwork (Hayes, 2014), frequency of collaboration may also contribute to perceived teamwork quality and was therefore included in the latent factor. The latent factors physician professional experience, case clarity, and perceived teamwork quality were modeled separately for junior and senior physicians because they may differ between team members. In the latent regression model, we postulated that the perceived teamwork quality can be explained by the following latent predictors: *physician professional experience*, *case clarity*, *case urgency*, and *workload.* We allowed for correlations between all these predictors.

### 2.4. Ethics statement

Patient data were collected during usual care in the ED and internal medicine ward. No additional patient data were collected for this study. Physicians participated on a voluntary basis. Anonymity of participants, both patients and physicians, was maintained at all times by pseudonymizing physician and patient data. The local ethics committee of the Canton of Bern registered the study as a quality assessment study under KEK No. 197/15 and waived the need for informed consent. The study protocol was previously published (Hautz et al., 2016a).

## 3. Results

In total, 55 physicians took part in the study and provided 644 questionnaires for 495 patients (65.6% of the total study population of the cDx study; for detailed patient demographics see Supplementary Table S1). For 149 patients, two questionnaires were available, filled in by the junior and senior physician; for 346 patients, only one questionnaire from either the junior or the senior physician was available.

### 3.1. Descriptive results

Of the 50 participating physicians in the final sample, 35 were junior physicians (*M*_age_ = 31.1 years, 60.0% female, mean postgraduate work experience 3.88 years) and 15 were senior physicians (*M*_age_ = 40.7 years, 53.3% female, mean postgraduate work experience 11.07 years). Descriptive characteristics for participating physicians are shown in Table 2.

**Table 2:** Descriptive statistics for participating physicians (*N* = 50).

Physician characteristic	Junior physicians	Senior physicians
*N* (%)	35 (70.0)	15 (30.0)
Age (years), mean (SD)	31.1 (2.67) (37% missing)	40.7 (3.73) (75% missing)
Gender, *n* (*%*)		
Male	13 (37.1)	6 (40.0)
Female	21 (60.0)	8 (53.3)
Unknown	1 (2.8)	1 (6.7)
Work experience total (years), mean (SD)	3.88 (1.63) (22.9% missing)	11.07 (2.86) (53.3% missing)
Work experience in ED (years), mean (SD)	1.33 (1.12) (22.8% missing)	4.93 (2.95) (53.3% missing)

ED, emergency department.

### 3.2. Questionnaire analyzes

Junior physicians provided questionnaires for 414 patients and senior physicians for 230 patients, with an overlap of 149 patients. As shown in Table 3, junior and senior physicians provided on average intermediate to high ratings concerning their confidence in their diagnoses, familiarity with similar cases, and the ease of the diagnostic process. In more than 70% of cases, junior and senior physicians rated their patients’ presentation as typical for the diagnosis made. With regard to teamwork, both junior and senior physicians rated their teamwork as of high quality (*M*_junior_ = 4.35, SD = 0.88, *M*_senior_ = 4.43, SD = 0.69) and the frequency of their collaboration as high (*M*_junior_ = 4.05, SD = 0.98, *M*_senior_ = 4.12, SD = 0.91). No significant differences between junior and senior physicians in any of these ratings were revealed (Table 3).

**Table 3:** Descriptive statistics of questionnaire data.

Measure	Questionnaires of junior physicians	Questionnaires of senior physicians	*t*-test results
	*M* (SD)	*M* (SD)	
Confidence in diagnostic accuracy	3.88 (1.18)	3.82 (0.95)	*t*(560) = −0.644, *p* = 0.520
Ease of the diagnostic process	3.48 (1.22)	3.43 (1.02)	*t*(548) = −0.585, *p* = 0.559
Familiarity with a case	3.94 (1.07)	4.03 (0.93)	*t*(528) = 1.099, *p* = 0.272
Case is typical	0.70 (0.46)	0.72 (0.45)	*t*(480) = 0.507, *p* = 0.613
Quality of teamwork	4.35 (0.88)	4.43 (0.69)	*t*(571) = 1.35, *p* = 0.178
Frequency of collaboration	4.05 (0.98)	4.12 (0.91)	*t*(501) = 0.891, *p* = 0.373

*N* = 414 junior physician questionnaires; *N* = 230 senior physician questionnaires. No missing data.

### 3.3. Latent regression model

**Figure 1:**
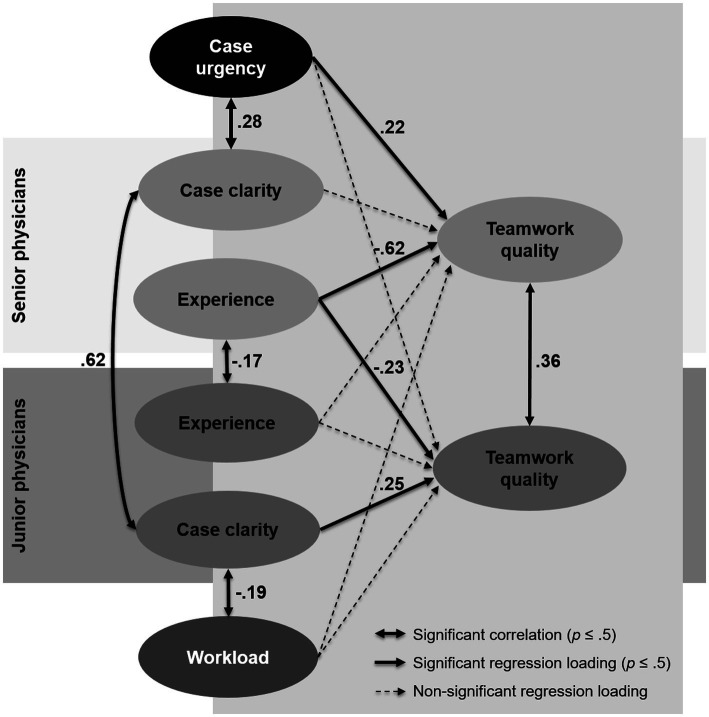
Relations of patient, physician, and context factors, as analyzed with a structural equation model.

The final SEM (Figure 1) revealed a very good model fit (*N* = 495; χ^2^ = 387.787; df = 244; *p* < 0.001; CFI = 0.953; RMSEA = 0.035). Whereas in the measurement models, all regression loadings were significant (*p* < 0.011), in the structural part of the model, only some of the latent predictors significantly contributed to the prediction of perceived teamwork quality. For the sake of clarity, only the significant correlations between predictors are shown in Figure 1.

The results showed that junior and senior physicians largely agreed in their ratings of case clarity (*r* = 0.62). For senior physicians, among the predictors, only their own work experience and case urgency significantly contributed to the prediction of their perceived teamwork quality (*R*^2^ = 44.5%). Whereas case urgency was positively related to the perceived teamwork quality (*r* = 0.22), we found a negative regression coefficient (*r* = −0.62) for the experience of the senior physician, meaning that higher experience of the senior physician negatively affected the teamwork quality perceived by him or her.

For junior physicians, their perceived teamwork quality could be explained by the experience of the collaborating attending physician and their own perceived case clarity, whereas all other predictors were not significant (*R*^2^ = 16.5%). Specifically, the experience of senior physicians was also negatively associated with the teamwork quality perceived by the junior physicians (*r* = −0.23), but this relation was less pronounced than it was for senior physicians. Moreover, for junior physicians, we found a significant positive regression coefficient for case clarity (*r* = 0.25), meaning that higher case clarity was related to higher perceived teamwork quality.

After the latent regression, a positive residual correlation still remained between the latent factors perceived teamwork of senior physicians and the perceived teamwork of junior physicians (*r* = 0.36), meaning that these two factors still had some variance in common that was not covered by the predictors included in the model. Another interesting result was that for the senior physicians, case urgency was positively related to perceived case clarity (*r* = 0.28), whereas for junior physicians, the perceived case clarity was negatively correlated with objective workload (*r* = −0.19). Complete results can be seen in the Mplus output file in our OSF repository (Kämmer et al., 2022).

## 4. Discussion

“Teamwork [is] in the eye of the beholder” (Makary et al., 2006, p. 746)–a number of studies have revealed, and these teamwork perceptions are an important factor that may influence individual, team, and organizational effectiveness (Dechairo-Marino et al., 2001; Manser, 2009; Kristensen et al., 2015; Berry et al., 2020). We have extended previous research by not only exploring perceptions of teamwork quality in a team setting that is common in many educational settings, namely, the team of trainee (i.e., junior physician) and supervisor (i.e., senior physician) diagnosing and treating patients in the ED, but also using SEM analyzes to investigate the role of situational and stable factors underlying these perceptions to generate new hypotheses. Also, we provide insights based on field data, thus addressing the demand for more studies of teams “in the wild” (Salas, 2008).

Our explorative analyzes of survey field data revealed highly positive evaluations of teamwork by supervisors *and* trainees. Additional SEM analyzes showed that these perceptions were mainly driven by the case-specific clarity and urgency, and the supervisor’s work experience.

The situational factors that determined perceived teamwork quality were case clarity and case urgency: the clearer the diagnosis (for trainees) and the more urgent the treatment (for supervisors), the better was perceived teamwork. We suspect that both these aspects helped the team know what to do and thus facilitated coordination between members and hence (perceived) teamwork quality. For case urgency, the rationale is that patients who arrive in critical condition and require urgent treatment often have more pronounced symptoms and are treated according to specific medical algorithms, such as when resuscitation is required. Also, at least in the ED under investigation, critically ill patients are always initially examined and treated jointly by the junior and senior physician together. In these situations, the junior is closely supervised and decisions regarding patient management are made directly at the bedside by the team. This may result in less cognitive load and a clearer coordination process compared to treating less urgent patients with, for example, nonspecific symptoms. The positive correlation between case clarity and case urgency (for senior physicians) supports this explanation. In contrast, less critically ill patients are—for educational reasons—usually initially assessed by the junior physician alone, who then discusses the case with the senior physician (who is always responsible for the final decisions). This often requires a more complex clinical reasoning process on behalf of the trainee and the supervisor. More generally, we would suggest that these observations indicate a moderating effect of task type and complexity on *perceived* teamwork quality. Follow-up research on this notion would extend prior theoretical and empirical work on the impact of the task type on *objective* team performance and behavior (Antoni and Hertel, 2009; Tschan et al., 2011; Schmutz et al., 2015).

Interestingly, although supervisors and trainees largely agreed in their judgment of case clarity, trainees’ judgments were negatively impacted by workload, but not supervisors’ judgments. In other words, trainees judged a case to be less clear, the louder and more crowded it was around them. We would thus hypothesize that everything that helps render the case clearer and reduces the cognitive load, such as clear instructions, reduced noise, structured communication with the supervisor, or feedback, may facilitate teamwork for trainees.

The stable factor that determined perceived teamwork quality (negatively) was the supervisor’s work experience: the more experienced the supervisor was, the less positive was perceived teamwork quality, for both trainees and (to a larger extent) supervisors. One explanation for this could be a greater professional disparity as a consequence of more work experience. With more experience, senior physicians may develop higher expectations of their trainees concerning what the trainees should know and do, and more nuanced conceptualizations of teamwork. If these expectations are then not met (from the perspective of the senior physician) or are perceived as too high (from the perspective of the junior physician), this could have a negative impact on the assessment of the quality of collaboration of both parties. Also, it is known from studies with interprofessional teams that different rationalities and priorities of team members may result in communication and coordination problems (Kvarnström, 2008; Rydenfält et al., 2012). To explore this question further, future research should investigate supervisors’ and trainees’ expectations of each other and their conceptualizations of teamwork, their respective roles, and tasks (Sebok-Syer et al., 2018; Rydenfält et al., 2019). If these turn out to be very different, informing both parties about each other’s expectations may help decrease misunderstandings and increase mutual empathy (Ebert et al., 2014) and ultimately enhance individual and team effectiveness.

Taken together, our findings have methodological, theoretical, and practical implications. On theoretical grounds, our findings shed light on the complex structure underlying perceptions of teamwork quality, which, at the same time, call for further research. Methodologically, the finding that the same factors may affect supervisors’ and trainees’ ratings of teamwork to different degrees suggests caution when attempting to compare or aggregate team members’ ratings of teamwork quality, even when ratings of teamwork do not differ in their numerical value (Tscholl et al., 2015; Sebok-Syer et al., 2018). Practically, the same finding is informative for educators and practitioners who need to decide on the necessity of organizing a debriefing or after-action review (Jarrett et al., 2016; Weiss et al., 2017) after a team event or on ways to (re)design workplace-based settings and processes; given our findings, it seems indicated to collect ratings of all participating members and not just those of the seniors or leaders (see also Hautz, S. C. et al., 2020).

### 4.1. Limitations

This study has several limitations. First, our results are based on cross-sectional data and therefore do not allow for causal interpretations. Second, because the ratings of teamwork quality were in general very positive, it is likely that there is a ceiling effect and thus the variance of those ratings is shrunk. This, in turn, may have led to an underestimation of the parameters in the regression analysis. Third, additional contextual and relationship factors such as psychological safety, team cohesiveness (Bravo et al., 2019), trust (Wang et al., 2019), autonomy (van Mierlo et al., 2006), cognitive load (Durning et al., 2012), and leadership style may also affect perceptions of teamwork and should be measured in future studies (Olson et al., 2020). Fourth, a limitation can be seen in our use of a two-item measure to capture teamwork quality. Despite its advantage of being short and despite evidence of the general suitability of single-item measures to capture overall concepts (Postmes et al., 2013; Müller et al., 2018), our measure may have captured only the collaboration dimension of teamwork, excluding other processes such as coordination or communication (Hoegl and Gemuenden, 2001; Rousseau et al., 2006; Reeves et al., 2018). In other contexts where time is less scarce than in an ED, it might be feasible to use longer measurement tools of teamwork quality (e.g., Shortell et al., 1991; Hoegl and Gemuenden, 2001; Temkin-Greener et al., 2004; Keebler et al., 2014) to understand how the method used to measure teamwork quality impacts results. It seems unlikely, however, that the interpretation of what a certain item is intended to measure varies systematically between supervisors and trainees. We would thus argue that this limitation can hardly cause the effects observed here.

### 4.2. Conclusion

To improve workplace culture and team effectiveness, research into the attitudes, perceptions and evaluations of personnel is relevant (c.f. Ummenhofer et al., 2001). For understanding the dynamics of work environments, it is important to take into account the organizational, physical and social context factors under which teams work (Reason, 2000). Here, we examined stable and situational factors that may influence team members’ assessments of teamwork quality when collaborating in a high-risk setting. Three factors turned out to have a major impact on teamwork perceptions, though to different degrees, depending on the role of the member as either supervisor or trainee: case clarity, case urgency, and supervisor’s work experience. Our insights into the complex underpinnings of teamwork perceptions may be informative for organizational, educational, and research endeavors targeting improved teamwork.

## Data availability statement

Original questionnaires are available under https://doi.org/10.17605/OSF.IO/JTUQ7. Complete results can be retrieved from our OSF repository https://doi.org/10.17605/OSF.IO/PYQ48. Data are available upon request from the corresponding author to researchers eligible to work with codified personal health care data under Swiss legislation. Eligibility will be determined by Kantonale Ethikkomission Bern when needed.

## Ethics statement

The ethics committee of the Canton of Berne registered the study as a quality evaluation study under No. 197/15 and waived the requirement for informed patient consent. All patients provided a general consent for the use of their data according to Swiss law.

## Author contributions

JEK, SE, OK-H, and WEH conceived and designed the study. WEH, SW-S, SCH, TB, and TCS collected the data. JEK and OK-H performed the statistical analyzes. JEK, SE, and WEH drafted and revised the manuscript. All authors reviewed and approved the final manuscript and have agreed to be accountable for all aspects of the work.

## Funding

JEK has received funding from the European Union’s Horizon 2020 research and innovation program under the Marie Skłodowska-Curie grant agreement no. 894536, project “TeamUp.” TCS holds the endowed professorship for emergency telemedicine at the University of Bern established by the Touring Club Switzerland. The sponsor had no influence on the content of the research or the decision to publish. TB and CS received funding from the European Union’s Horizon 2020 research and innovation program under the grant agreement no. 101021775, project “Med1stMR.” This study was in part funded by a research grant no. 407740_187284 of the Swiss National Science Foundation to WEH for the project “The digital diagnostician: How information technology affects medical diagnoses.”

## Conflict of interest

WEH has received research funding from the European Union, the Swiss National Science foundation, the Zoll Foundation, Dräger Medical Germany, Mundipharma Research United Kingdom, MDI International Australia, and Roche Diagnostics Germany, all outside the submitted work. WEH has provided paid consultations to the AO Foundation Switzerland and MDI International Australia, all outside the submitted work. WH has received financial support for a conference he chaired from EBSCO Germany, Isabel Healthcare United Kingdom, Mundipharma Medical Switzerland, and VisualDx United States, all outside the submitted work.

The remaining authors declare that the research was conducted in the absence of any commercial or financial relationships that could be construed as a potential conflict of interest.

## Publisher’s note

All claims expressed in this article are solely those of the authors and do not necessarily represent those of their affiliated organizations, or those of the publisher, the editors and the reviewers. Any product that may be evaluated in this article, or claim that may be made by its manufacturer, is not guaranteed or endorsed by the publisher.
