# Supplementary material for: What factors affect team members’ evaluation of collaboration in medical teams?
Source: Front Psychol. 2023 Jan 12;13:1031902. doi: 10.3389/fpsyg.2022.1031902 (PMC9877456; doi:10.3389/fpsyg.2022.1031902)
Supplement: Supplementary file 1 [file Table_1.DOCX]

Supplementary Material

# Supplementary Table

**Table S1.** Descriptive statistics for patients included in analyses (*N* = 495)

| **Patient characteristic** | ***n* (%)** |
| --- | --- |
| Gender |  |
| Male | 272 (54.9) |
| Female | 223 (45.1) |
| Triage category^a^ |  |
| 1 | 27 (5.5) |
| 2 | 218 (44) |
| 3 | 230 (46.5) |
| 4 | 16 (3.2) |
| 5 | 4 (0.8) |
| Treated in resuscitation bay^b^ | 50 (10.1) |
| Death | 23 (4.6) |

*Note*. Mean age of patients = 66 years, *SD* = 18.

^a^ Swiss Triage System categories; see Table 1. ^b^ Resuscitation bay: Acute treatment place for patients.
